# Clinical and biological significance of miR-23b and miR-193a in human hepatocellular carcinoma

**DOI:** 10.18632/oncotarget.14332

**Published:** 2016-12-28

**Authors:** Ilaria Grossi, Bruna Arici, Nazario Portolani, Giuseppina De Petro, Alessandro Salvi

**Affiliations:** ^1^ Department of Molecular and Translational Medicine, Division of Biology and Genetics, University of Brescia, Brescia, Italy; ^2^ Department of Clinic and Experimental Sciences, Surgical Clinic, University of Brescia, Brescia, Italy

**Keywords:** microRNAs, epigenetics, 5-Aza-2’-deoxycytidine, HCC, molecular targets

## Abstract

Hepatocellular carcinoma (HCC) is the most common cancer of the liver with a very poor prognosis. The dysregulation of microRNAs (miRs) is indeed implicated in HCC onset and progression. In this study, we have evaluated the expression of miR-23b and miR-193a in a large cohort of 59 and 67 HCC patients, respectively. miR-23b and miR-193a resulted significantly down-regulated in primary HCCs compared to their matched peritumoral counterparts. Furthermore, patients with higher miR-193a expression exhibited longer OS and DFS, suggesting that miR-193a may be a molecular prognostic factor for HCC patients. Since the regulation of miRs by DNA methylation may occur in human cancers, we verified whether the down-modulation of miR-23b and miR-193a in HCC tissues could be related to DNA methylation. An inverse trend between miR-23b expression and DNA methylation was observed, indicating that miR-23b can be epigenetically regulated. By contrast, the down-regulation of miR-193a was not mediated by DNA methylation. To verify the potential role of miR-23b and miR-193a as responsive molecular targets *in vitro*, we used the inhibitor of DNA methylation 5-aza-dC to restore miR-23b expression level in combination with miR-193a transfection. The combined treatment led to a significant inhibition of cellular proliferation and migration. Taken together, our findings provide evidence that miR-23b and miR-193a may be molecular diagnostic and prognostic factors for HCC; furthermore, miR-23b and miR-193a are responsive molecular targets for limiting HCC cell aggressiveness in combination with the epigenetic drug 5-aza-dC. Moreover, our results provide new advances in the epigenetic regulation of these miRs in HCC.

## INTRODUCTION

Hepatocellular carcinoma (HCC) is the most common type of primary liver cancer and it accounts for 80-90% of total cases. HCC represents the third leading cause of cancer death worldwide and due to poor prognosis and high incidence, it represents a malignancy of global importance [1, 2]. To date, surgical resection and orthotopic liver transplantation are considered the only possibly curative therapies for early stage HCC. However, only one third of patients are eligible for these therapies and long term results are not completely satisfactory [3, 4]. For patients with inoperable HCC, percutaneous ablation and chemoembolization are widely used as loco-regional therapeutic strategies. The sole systemic treatment available against advanced HCC is the oral multikinase inhibitor sorafenib that prolongs the overall survival (OS) of HCC patients from 7.9 to 10.7 months [5, 6]. However, some patients develop resistance to sorafenib suggesting that innovative molecular therapies are urgently needed.

In the last decade, several data from high throughput analyses have delineated the landscape of genetic alterations of HCC, providing important insights into the characterization of possible biomarkers and targets in order to improve prognosis of HCC patients. However, actually, none of this knowledge is translated into clinical practice [7]. Consequently, a more in-depth understanding of HCC onset and progression is paramount to identify responsive molecular therapeutic targets.

Similarly, with other tumors, the pathogenesis of HCC is a multistep process that starts from pre-neoplastic lesions that progress in HCC. The exposure to risk factors that determine or aggravate the chronic inflammation of the liver contributes to the accumulation of genetic and epigenetic alterations. This may result in activation of oncogenes, silencing of tumor suppressors genes, in microRNAs and in other non-coding RNAs dysregulation [8, 9]

MicroRNAs (miRNAs) are a class of endogenous, small non-coding RNAs (19-22 nucleotides in length) that regulate gene expression at post-transcriptional level, playing an important role in physiological processes, such as differentiation, apoptosis and cell growth. Several studies demonstrated that miRNAs are dysregulated in many types of cancers and contribute to tumorigenesis functioning both as tumor suppressor genes both as oncogenes (OncomiRs). Additionally, molecular approaches based on miRNAs may constitute a promising strategy for the treatment of solid tumors by relying on the ability of miRNAs to target different oncogenes at the same time [10, 11]. In recent years, the epigenetic regulation of miRNAs has been reported for many types of cancers. The enhancement of methods for measuring DNA methylation at specific genomic loci allowed the identification of DNA hyper-methylation of CpG sites within CpG islands as a mechanism involved in the down-regulation of miRNAs [12–14]. Interestingly, Kozaki and colleagues found that 11.6% (122/1048) of miRNAs were epigenetically regulated in 23 cancer types and that 19.5% of miRNA coding sequences had a CpG island within 5 kb upstream [15]. In 2008 Datta *et al*. individuated the first miRNA silenced by hypermethylation in HCC cells and tissues, miR-1 [16]. They also demonstrated that the ectopic expression of miR-1 in HCC cells reduced their aggressive properties and that the treatment with hypomethylating agent 5-Azacytidine induced the down-regulation of the miRNA targets FoxP1, MET and HDAC4. Subsequently also miR-129-2, miR-124, miR-203, miR-125b, miR-34b and miR-200b were recognized as novel tumor suppressor miRNAs epigenetically silenced in HCC [17–21].

In the context of microRNA and HCC, our previous results identified miR-193a as a negative regulator of urokinase type plasminogen activator (uPA) [22] and miR-23b as a negative co-regulator of uPA and MET (a receptor tyrosine kinase, RTK) [23], that are considered both unfavourable prognostic factors in HCC patients [24]. In this study, we first examined the expression of miR-23b and miR-193a in a large number of HCC specimens to explore their possible role as diagnostic and prognostic tissue molecular markers. Next, since bioinformatics analysis showed the presence of CpG islands near the coding sequences of these miRNAs we verified whether the DNA methylation was involved in the regulation of miR-23b and miR-193a expression in HCC. Recent studies aimed at the development of anti-cancer molecular approaches based on epigenetic drugs, included the inhibitor of DNA methylation 5-Aza-2’-deoxycytidine (5-aza-dC, Decitabine) that has been approved in the treatment of haematological malignancies and its effects on solid tumors were worth considering [25]. Since we found that DNA methylation was involved in the down-regulation of miR-23b, but not of miR-193a, we tested the effects of 5-aza-dC that restored miR-23b expression, in combination to miR-193a transfection in HCC cells.

## RESULTS

### miR-23b and miR-193a are down-regulated in human primary HCCs

**Figure 1:**
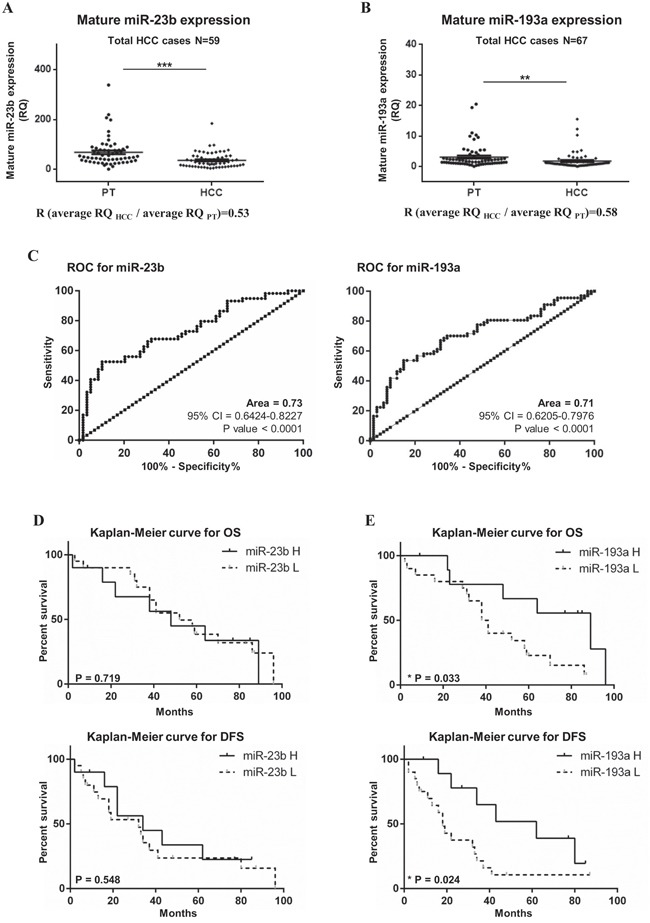
Expression of mature miR-23b and miR-193a in HCC and peritumoral specimens The expression of miR-23b and miR-193a was detected separately by stem-loop qPCR in 59 and 67 HCC patients, respectively, from both HCC tissues and PT samples. The expression of mature miR-23b **A**. and miR-193a **B**. was generally lower in HCCs compared to corresponding PT counterparts. Statistical significance was determined by paired t-test analysis ***p<0.001 and **p<0.01. **C**. ROC analysis for the ability of miR-23b and miR-193a expression to discriminate between HCC and PT tissues. Kaplan-Meier analysis was performed for overall survival (OS) and disease free survival (DFS) based on the expression miR-23b **D**. and miR-193a **E**. where miR-H indicates high miR expression groups while miR-L indicates the low miR expression group.

We examined the expression of mature miR-23b and miR-193a in human HCC specimens and their peritumoral (PT) counterparts by real-time qPCR. In 59 HCC patients, the expression of mature miR-23b was significantly lower in HCC specimens compared with PT tissues from the same patients (average RQ_PT_ = 68.63 ± 7.50, average RQ_HCC_ = 36.60 ± 4.08, p<0.001; R [RQ_HCC_/ RQ_PT_]= 0.53; Figure 1A). Even though the overall expression of miR-23b was lower in HCC tissue compared to PT tissue, there were patients showing different trends. In particular, 38 HCC cases (64%) showed miR-23b down-regulation (R<0.7) while 8 HCC cases (14%) displayed up-regulation of miR-23b in HCC samples with respect to PT counterparts (R>1.3). Conversely, 13 patients (22%) showed an equal expression of miR-23b when HCC and PT tissues were compared (0.7<R<1.3; Supplementary Figure 1A). Among the 67 HCC patients tested, miR-193a resulted significantly down-regulated in HCC compared to PT tissues (average RQ_PT_ = 3.13 ± 0.47; average RQ_HCC_ = 1.80 ± 0.33; p<0.01; R [RQ_HCC_/ RQ_PT_] = 0.58; Figure 1B). As for miR-23b, individual patients showed different trends. We found that miR-193a was down-regulated in 43 HCC cases (64%) while it was up-regulated in 14 HCC cases (22%) compared with corresponding PT tissues. Ten cases (10/67, 15%) had no change in miRNA expression level between HCC and PT tissues (Supplementary Figure 1B). To evaluate the correlation between miR-23b and miR-193a expression and clinical pathological characteristics, the patients were divided into 2 groups with high (R>0.8) and low (R<0.8) miR expression. However, non-significant correlations were observed between miRs expression and clinical and pathological characteristics (Supplementary Table 1). ROC curve and Kaplan-Meier analysis were performed to prove the diagnostic and prognostic significance of miR-23b and miR-193a in HCC. Areas under ROC curve (AUC) of 0.73 (95% CI = 0.64-0.82; p<0.001) and 0.71 (95% CI = 0.62-0.80; p<0.001) were obtained for miR-23b and miR-193a, respectively (Figure 1C). These results suggested that the expression of miR-23b and miR-193a may discriminate HCC from PT samples with fair accuracy in the cohort examined. To conduct the Kaplan-Meier analyses on the 30 cases available, we divided HCC samples into group with high miR expression (R>0.8) and low miR expression (R<0.8). No significant difference was reported in the OS and the disease-free survival (DFS) were found between cases with higher miR-23b expression compared with lower expression (Figure 1D). Conversely, the group with higher miR-193a expression had a significantly increased OS and DFS than the group with down-regulated miR-193a. In fact, the higher miR-193a expression group median OS was of 89 months compared with 39.5 months of the group with lower miR-193a expression (Log-Rank test p<0.05). The median DFS for the group of patients with higher miR-193a expression was of 62 months while the same value for the group with lower miR-193a expression was of 18 months (Log-Rank test p<0.05; Figure 1E).

### DNA methylation is involved in the regulation of miR-23b expression in primary HCC

**Figure 2:**
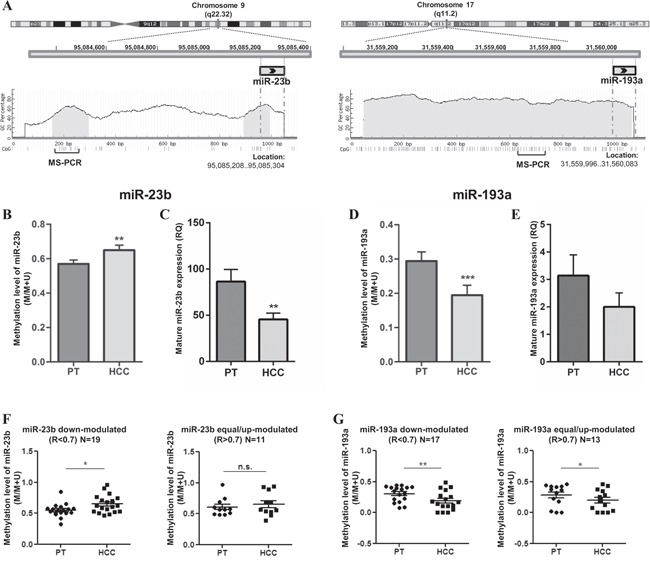
DNA methylation levels of miR-23b and miR-193a and relative expression in primary HCCs (N=30) **A**. Schematic illustration of chr 9 and chr 17 where miR-23band miR-193a coding sequences are located, respectively. CpG islands in light grey and relative GC percentage were shown in a region spanning 1,000 bp upstream of each miR. Each small vertical bar indicates a single CG dinucleotide and the regions examined by MS-PCR are represented below.DNA methylation and miRs expression levels are reported for miR-23b **B-C**. respectively and miR-193a **D-E**. respectively. Histograms indicate the average level of methylation detected with MS-PCR or the mean RQ obtained in qPCR while bars show SEM. (B) The methylation level of miR-23b was significantly higher in HCC than in PT tissues while the expression level was lower (C), indicating an inverse trend between DNA methylation and miRNA expression.For miR-193a, both methylation (D) and expression (E) levels were decreased in HCC specimens respect to PT counterparts. **F**. In the group of HCCs displaying miR-23b down-regulation, the methylation level was significantly higher than in PTs; no difference in DNA methylation was observed in the group of HCCs with equal or up-modulation of the miRNA. **G**. For miR-193a, DNA methylation level was decreased in HCC samples in both down-modulated and up-modulated groups. *p<0.05, **p<0.01, ***p<0.001 in paired t-test analysis.

In order to understand whether the modulation of miR-23b and miR-193a in HCC tissues was mediated by DNA methylation, we compared the level of DNA methylation and miRs expression in 30 HCC cases more recently collected. DNA methylation level was detected by MS-PCR (Supplementary Figure 2) and mature miRs expression was examined by qPCR. As shown in Figure 2A, miR-23b coding sequence was preceded by two CpG islands (141bp and 101bp in length, respectively) in a 1.0kb upstream region while miR-193a coding sequence is embedded in a CpG island (1,477bp in length). The results obtained in MS-PCR and qPCR showed an inverse trend between DNA methylation level and miR-23b expression. In fact, average methylation level was significantly higher (p<0.01; Figure 2B) and miR-23b expression was significantly lower (p<0.01; Figure 2C) in HCC tissues compared with PT tissues. On the contrary, the average methylation level of miR-193a at CpG sites resulted significantly reduced in HCC respect PT counterparts (p<0.001; Figure 2D) with the miRNA generally down-regulated in this group of HCC cases (Figure 2E). To analyze further, we divided the HCC samples into two groups according to the expression of each miRNA where R<0.7 indicated miRNA down-modulation and R>0.7 indicated that the miRNA was equal- or up-modulated in HCC compared to PT tissues. Interestingly, the average methylation level of miR-23b in the first group was significantly higher in HCC than PT tissues (p<0.05) while no difference was observed in the second group (Figure 2F). On the contrary, methylation level of miR-193a was significantly lower in HCCs, uncoupled with miRNA expression level (Figure 2G). These findings suggested that the methylation of CpG sites examined was involved in the down-regulation of mature miR-23b in primary HCC, but not in miR-193a down-regulation. However, the lack of an association between DNA methylation and miR-23b expression in the HCC group with up-modulated miRNA hinted at the presence of additional mechanisms taking part in the regulation of miR-23b in human HCCs and probably also in miR-193a regulation.

### Treatment with 5-aza-dC restored miR-23b expression in cultured HCC cells

**Figure 3:**
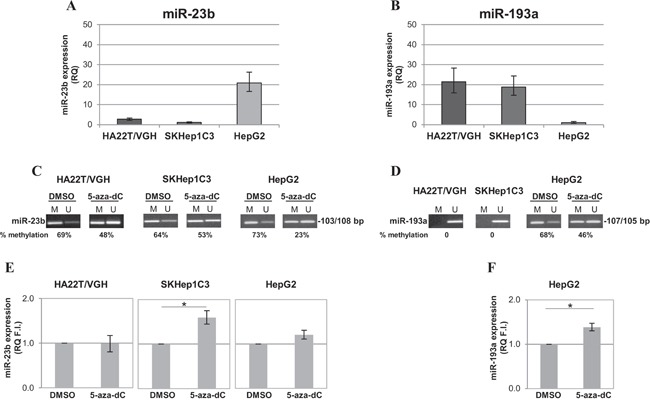
Expression and methylation levels of miR-193a and miR-23b in HCC cell lines **A-B**. miR-23b and miR-193a expression levels, respectively, were detected by stem-loop qPCR in human HCC cells. Histograms represent the mean RQ while bars represent RQ maximum and minimum values. **C**. MS-PCR analysis showed that miR-23b CpG sites resulted partially methylated and the treatment with 5-aza-dC led to their demethylation. **D**. miR-193a CpG sites were completely unmethylated in undifferentiated HCC cells and partially methylated in HepG2. The treatment with 5-aza-dC induced the demethylation of analysed site in HepG2. The percentage of DNA methylation (IOD M/IOD M + IOD U × 100) is indicated for each sample. **E-F**. 5-aza-dC treatment significantly increased mature miR-23b expression in SKHep1C3 and mature miR-193a expression in HepG2, respectively; mean of three experiments ± SEM are reported; *p<0,05 using unpaired t-test analysis.

Since the over-expression of miR-23b decreased proliferation and migration abilities of HCC cells [23], we evaluated whether treatment with the inhibitor of DNA methylation 5-aza-dC could induce up-regulation of miR-23b in HCC-derived cells. Firstly, the expression levels of miR-23b and miR-193a were examined in different HCC cell lines. Stem-loop qPCR revealed that miR-23b was detected at low level in undifferentiated HCC cells (HA22T/VGH and SKHep1C3) and it was moderately expressed in differentiated HepG2 cells (Figure 3A). On the contrary, miR-193a expression level was moderate in HA22T/VGH and SKHep1C3 and very low in HepG2 cells (Figure 3B). Regarding the level of DNA methylation, miR-23b CpG sites resulted partially methylated in HCC cells as detected by MS-PCR. In particular, the band specific for methylated sequence was predominant with a percentage of methylation equal or greater than 60% in all HCC cell lines (Figure 3C). Furthermore, miR-193a CpG sites resulted completely unmethylated in HA22T/VGH and SKHep1C3 cells and 68% methylated in HepG2 cells (Figure 3D). Treatment with 5-aza-dC reduced the methylation level of miR-23b CpG sites in all cell lines but particularly in HA22T/VGH and HepG2 cells. However, the reduction of methylation in HA22T/VGH and HepG2 cells did not lead to a significant difference in miR-23b expression (Figure 3E). Interestingly, SKHep1C3 cells showed only a modest reduction in methylation but were evidently more sensitive to demethylation since mature miR-23b expression significantly increased (p<0.05) in cells after treatment with the inhibitor of DNA methylation compared with untreated cells (Figure 3E). Similarly, 5-aza-dC treatment in HepG2 cells led to the decrease in methylation level of miR-193a CpG sites (Figure 3D) and 39% increase in mature miR-193a expression (p<0.05; Figure 3F). All together, these results showed that the methylation of DNA contributed to the regulation of miR-23b in SKHep1C3 cells and miR-193a in HepG2 cells.

### Effects of miR-193a ectopic expression and 5-aza-dC treatment on expression of MET and uPA

**Figure 4:**
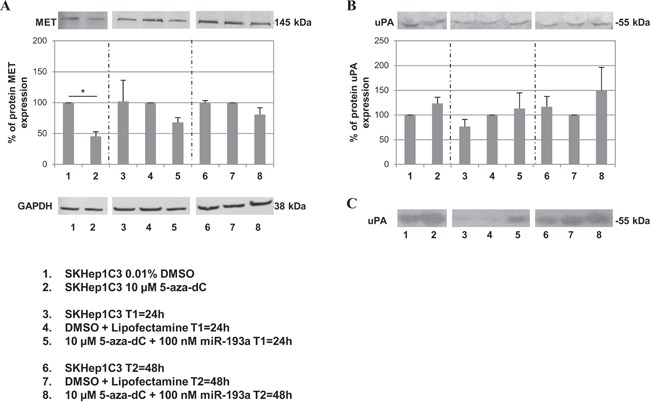
Effects of combined treatment with 5-aza-dC and miR-193a on MET and uPA protein expression levels SKHep1C3 cells were treated with DMSO (vehicle) and 10 μM 5-aza-dC for 5 days (lanes 1 and 2) or treated with 10 μM 5-aza-dC in combination to 100 nM miR-193a transfection (lanes 5 and 8) and relatives controls (lanes 3, 4, 6 and 7) at 24h and 48h from transfection. Western blot was performed to detect MET and GAPDH expression **A**. on cell extracts and uPA expression **B**. and corresponding enzymatic activity **C**. on conditioned medium. The histograms represent the mean IOD (Integrated Optical Density) values in percentage while bars are SEM. *p<0.05 using unpaired t-test analysis.

Since 5-aza-dC treatment induced the expression of miR-23b in SKHep1C3 cells, we hypothesized that by combining the inhibitor of DNA methylation with miR-193a transfection it could be possible to re-establish the homeostatic level activities of miR-23b and miR-193a. In our previous published studies [22, 23] we have demonstrated that miR-23b and miR-193a negatively regulated the expression of uPA and MET in HCC cells. For this reason we examined whether the combined use of 5-aza-dC and miR-193a transfection might alter the expression of MET and uPA. For this purpose, MET and uPA protein expression was evaluated by western blot in SKHep1C3 cells treated with 10 μM 5-aza-dC alone or in combination with 100 nM miR-193a. The receptor tyrosine kinase MET is synthesized as a precursor protein of 170 kDa that is cleaved to yield a 50-kDa α-chain and a 145-kDa β-chain linked together by disulfide bonds. After 5 days, there was a 2.2 fold decrease in the MET β-chain expression level (equivalent to 55%) in cells treated with 10 μM 5-aza-dC as compared with the control exposed to DMSO only (p<0.05; Figure 4A). The reduced expression of MET protein was also observed in cells treated with 10 μM 5-aza-dC and transfected with 100 nM miR-193a where 32% and 20% decrease at 24h and 48h after transfection, respectively, was observed when compared to cells treated with DMSO and lipofectamine alone. In contrast, uPA protein level was up-regulated by 23% after 5-aza-dC treatment and by 50% after co-treatment with 5-aza-dC and miR-193a at 48h from transfection, as compared with corresponding controls (Figure 4B). The corresponding enzymatic activity evaluated in zymography confirmed the trend of uPA protein expression (Figure 4C). To support the above mentioned results, we treated 2 more HCC cell lines (HepG2 and HA22T/VGH) with the DNA methylation inhibitor 5-aza-dC and miR-193a to verify whether uPA and MET expression may be affected. In HepG2 and HA22T/VGH cells the MET expression level decreased in 5-aza-dC treated cells and in the co-treated cells, in line with the results found in SKHep1C3 (Supplementary Figure 3A and Supplementary Figure 3C). uPA protein expression did not change in HepG2 following combined treatments (Supplementary Figure 3B) and its enzymatic activity was undetectable (data not shown); in HA22T/VGH cells uPA protein and its enzymatic activity resulted inhibited following 5-aza-dC and miR-193a combined treatment (Supplementary Figure 3D and Supplementary Figure 3E).

### 5-aza-dC treatment and miR-193a ectopic expression decreased the proliferation and migration of HCC cells

**Figure 5:**
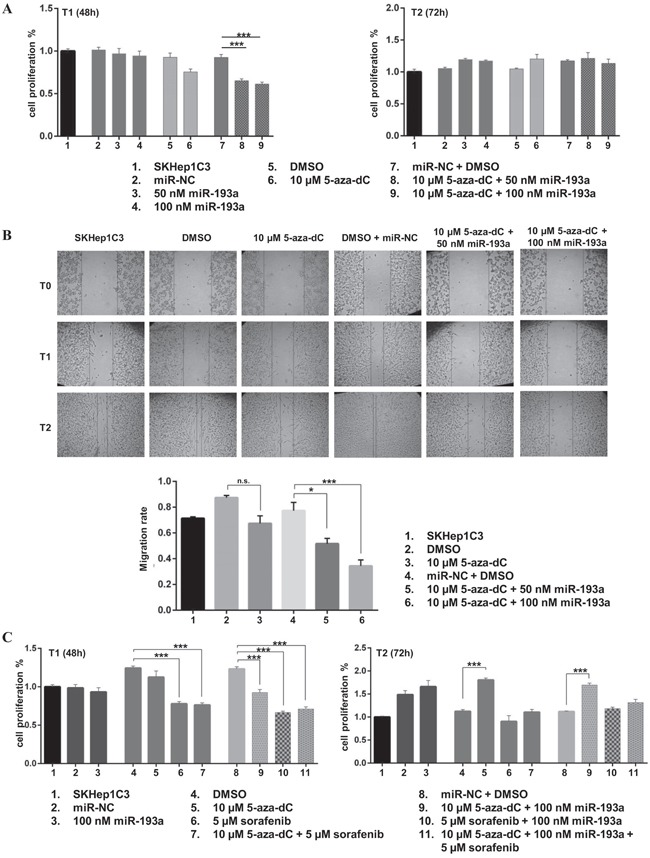
Ectopic expression of miR-193a in combination to 5-aza-dC treatment decreased proliferation and migration abilities of SKHep1C3 cells **A**. The MTT assay showed that the combined treatments with 5-aza-dC and miR-193a ectopic expression inhibited the growth of SKHep1C3 cells at T1 (48h) compared to controls and miRNA transfection alone. Histograms represent mean values of two experiments while bars represent SEM. *** p<0.001 in one-way ANOVA followed by Tukey test **B**. Scratch wound healing assay was performed to asses migration ability. Representative phase contrast images with magnification 10X at T_0_, T_1_ (20 hours) and T_2_ (30 hours) after wound are reported. Histograms represent the mean value of migration rate (scratch width T_0_ - scratch width T_2_/ scratch width T_0_); error bars are the SEM. ***p<0.001, *p<0.05 using one-way ANOVA followed by Tukey test. **C**. SKHep1C3 cells were treated with the indicated concentrations of 5-aza-dC, miR-193a and sorafenib, alone or in combination and the effects on cell proliferation were assessed by MTT assay. Results are representative of one of three experiments. The histograms represent averaged values of five replicates for each condition plotted as fold increased respect to SKHep1C3 cells; bars represent SEM. One-way ANOVA followed by Tukey test was used and ***p<0.001.

To determine the biological effects of 5-aza-dC treatment in combination with miR-193a ectopic expression, we pre-treated SKHep1C3 cells with 10 μM 5-aza-dC followed by transfection with synthetic pre-miR-193a at 50 and 100 nM doses and monitoring of cell growth, as described in the Materials and Methods section. The MTT assay data showed no significant difference of cell viability after miR-193a transfection, while the treatment with 5-aza-dC alone decreased cell growth by up to 18% at 48h after the last treatment as compared with untreated cells (Figure 5A). The combination of miR-193a transfection and 5-aza-dC treatment further significantly reduced cell proliferation. Particularly when 100 nM of miR-193a was used, the cell growth decreased up 32% (p<0.001) at 48h after transfection as compared with control cells (DMSO treated and miR-NC transfected).The inhibition of cell growth was not observed at 72h suggesting that 5-aza-dC treatment and miR transfection had a transient effect on proliferation. In these conditions, the expression levels of mature miR-23b was increased up to 72% following 5-aza-dC treatment and miR-193a transfection (Supplementary Figure 4A); miR-193a was upregulated up to 168 folds (Supplementary Figure 4B) after ectopic miR-193a transfection as verified by qPCR. Further, we evaluated the effects of 5-aza-dC and miR-193a transfection on SKHep1C3 cell migration. The treatment with 5-aza-dC alone decreased cell migration by 23% as compared with untreated cells (Figure 5B). The combination of miR-193a transfection and 5-aza-dC treatment further significantly reduced cell migration up to 56% (p<0.001) at 100nM miR dose concentration.

Subsequently, we verified whether a combined approach based on the co-administration of a low dose sorafenib (5 μM) in combination with 5-aza-dC or 5-aza-dC *plus* miR-193a could show a major inhibitor effect on HCC cell proliferation. As shown in Figure 5C, at T1 (48h) sorafenib alone displayed the highest inhibition of cell proliferation (37% decrease against DMSO alone, p<0.001) compared to miR-193a transfection and 5-aza-dC treatment. The combinations of 5-aza-dC *plus* sorafenib and sorafenib *plus* miR-193a significantly reduced cell proliferation ability compared to relative controls (39% and 46% decrease against DMSO and miR-NC + DMSO respectively; p<0.001). The same effect was observed after the co-administration of the 3 treatments (42% reduction, p<0.001) as compared with untreated cells (miR-NC + DMSO). After 72 hours (T2) from miR-193a transfection, only sorafenib maintained its inhibitory effect on cell proliferation, even if to a lower level (20% compared to DMSO). These findings suggested that the combination of 5-aza-dC treatment and miR-193a ectopic expression induced a considerable inhibition of proliferation and migration in HCC cells; furthermore the addition of sorafenib to 5-aza-dC, in presence or not of miR-193a, did not increase the inhibitory effects on cell viability carried out by the treatment with sorafenib alone.

### Effects of miR-23b and miR-193a ectopic expression on the proliferation and migration of HCC cells

**Figure 6:**
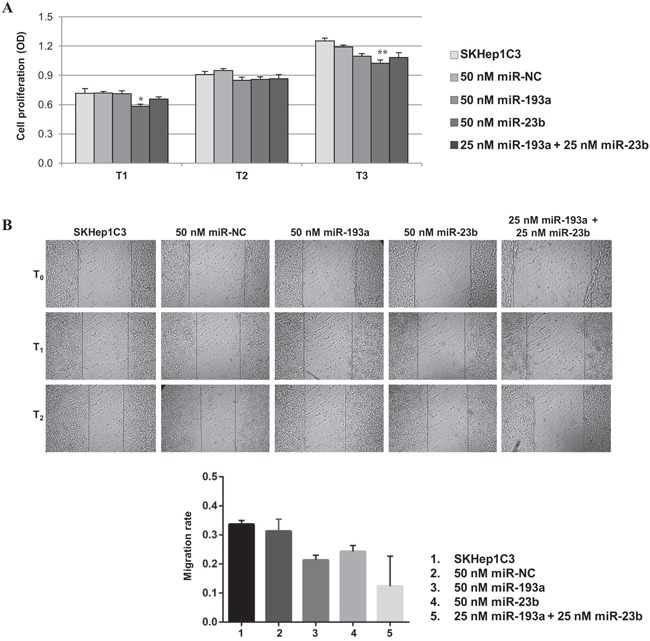
Effects of miR-23b and miR-193a ectopic expression on the proliferation and migration of SKHep1C3 cells MTT assay **A**. and scratch wound healing assay **B**. were performed in SKHep1C3 cells transfected with 50 nM of miR-NC, 50 nM of miR-193a, miR-23b, or a half dose of miR-193a + miR-23b. (A) Ectopic expression of miR-23b significantly decreased proliferation after 24 hours and 72 hours from transfection compared to controls. No additive effects were detected using miR-193a and miR-23b. The histograms represent averaged values of five replicates for each condition; bars represent SEM. One-way ANOVA followed by Tukey test was used and **p<0.01; *p<0.05. Results are representative of one of two experiments. (B) Representative phase contrast images with magnification 10X at T_0_, T_1_ (20 hours) and T_2_ (30 hours) after wound are reported. Histograms represent the mean value of migration rate (scratch width T_0_ - scratch width T_2_/ scratch width T_0_) for each sample; error bars are the SEM.

Since 5-aza-dC determines global changes in gene expression, we tested whether co-transfection of low doses miR-23b and miR-193a in SKHep1C3 cells may impair the cell proliferation and migration in order to compare the effects provoked by 5-aza-dC and miR-193a treatment. The combined transfection did not induce additive cell proliferation inhibition (Figure 6A). The major effect was observed 24 h after 50 nM miR-23b transfection (20%, p<0.05). The migration ability of SKHep1C3 was inhibited after miR-23b and miR-193a single transfection and an additive trend was found following the co-transfection with miRs (Figure 6B).

## DISCUSSION

In previously published studies, we have demonstrated that miR-23b directly targeted both MET and uPA in HCC cells and that the ectopic expression of miR-23b reduced proliferation and migration abilities of HCC cells [23]. More recently, we found that miR-193a negatively regulated uPA and the transfection of HCC cells with miR-193a decreased cell growth, increased apoptosis and sensitized the HCC cells to sorafenib [22]. In the present study carried out using a large cohort of patients with HCC, we found that miR-23b and miR-193a were significantly down-regulated in HCC specimens compared with peritumoral (PT) counterparts and the expression of both miRs could be used as a marker of diagnosis at molecular level. Moreover, the low expression of miR-193a resulted significantly related to reduced overall survival (OS) and disease free survival (DFS) of patients. The latter finding confirmed and complemented the results reported by Liu *et al*. that examined miR-193a expression in 95 formalin-fixed paraffin-embedded (FFPE) HCC tissues and corresponding PT [26].

In the recent years, epigenetic alterations have been highlighted as key factors during cancer development and progression; among which, aberrant methylation of CpG dinucleotides has been the best studied epigenetic modification in cancer, including HCC. Changes in DNA methylation level emerge at the early stage of hepatocarcinogenesis, affecting both coding and non-coding genes, such as genes encoding miRNAs [27, 28]. A number of miRNAs are found epigenetically silenced by DNA hypermethylation and their dysregulation usually resulted in the alteration of important pathways involved in carcinogenesis. Here, for the first time, we have found that DNA hyper-methylation was involved in the miR-23b down-modulation in HCC because we reported an inverse trend between DNA methylation and miR-23b expression in primary HCC. In line with this finding, miR-23b was found to be silenced by hypermethylation in prostate cancer tissues and cell lines [29], in glioma stem cells [30] and cervical cancer cell lines [31]. On the contrary, in the same HCC cases analysed the miR-193a down-modulation was not associated to a hyper-methylation of the miR-193a-related CpG island. Previous studies concerning miR-193a showed that it is regulated by DNA methylation in oral cancer and in non-small cell lung cancer [32, 33] and acute myeloid leukemia [34]. Therefore, we hypothesized that other mechanisms could take part in miR-193a regulation in HCC. Among these, Max and RXRα were reported as two transcription factors that bind directly miR-193a regulatory region and inhibit miR-193a expression in ER-Src transformed cells [35]. Furthermore, like protein-coding genes, miRNAs expression can be closely associated to histone modifications. Data obtained using miRNA microarray analysis showed that HDAC inhibitors modulated the expression of several miRNAs also in HCC cell lines [36, 37].

The next aim of the present work was the identification of an HCC *in vitro* model to modulate miR-23b and miR-193a expression. The analyses of DNA methylation level reported that miR-23b was methylated in all 3 HCC cell lines examined at low miR-23b expression level and miR-193a was methylated in differentiated HepG2 cells. On the contrary, in undifferentiated HCC cell lines, miR-193a resulted completely unmethylated. Previously, Ma *et al*. indicated that the 5-Fluorouracil-sensitivity of HCC cells, including HepG2, was related to the down-regulation of miR-193a mediated by DNA hypermethylation. They also showed the total demethylation of miR-193a in different HCC cell lines [38], in agreement with our results. In addition, in malignant pleural mesothelioma cells that displayed downregulation of miR-193a, this miR was detected unmethylated [39]. About the demethylating effects, we showed that miR-23b levels increased after treatment with the DNA methylation inhibitor 5-aza-dC in SKHep1C3 and in HepG2 cells. This increased sensitivity to inhibitor of DNA methylation coupled with the observation that DNA methylation levels of miR-23b and miR-193a were in line with those observed in HCC specimens supported our decision to use the SKHep1C3 cells to modulate miR-23b and miR-193a expression.

In recent years, the advances in the knowledge of epigenetics, suggested the use of epigenetic drugs for the treatment of solid tumors to obtain the demethylation of tumor suppressor genes and miRs at epigenomic level. Inhibitor of DNA methylations such as 5-Aza-2’-deoxycytidine and 5-Azacytidine have been approved for the treatment of myelodysplastic syndrome and their effects on apoptosis and senescence have been established in HCC cells [40, 41]. In order to define a design of a responsive experimental treatment approach based on the modulation of miR-23b and miR-193a, we attempted to restore miR-23b and miR-193a respectively by 5-aza-dC treatment and ectopic miR-193a expression in SKHep1C3 cells and we assessed the expression levels of the miR-23b and miR-193a targets, uPA and MET. Data obtained in western blot analysis conducted in SKHep1C3 cells and in 2 more cell lines (HepG2 and HA22T/VGH) were consistent with the hypothesis that global DNA demethylation downregulates MET through the activation of intracellular mechanisms, including increase of endogenous miR-23b. For example, it has been shown that DNA demethylation activates a fusion transcript LINE-1-MET that reduces the expression of MET in colon carcinoma cells [42]. Conversely, 5-aza-dC treatment with or without miR-193a transfection increased uPA protein levels in SKHep1C3 cells thus indicating that the ectopic expression of miR193a and the miR-23b upregulation induced by 5-aza-dC treatment had minor effects on uPA expression. However, in HA22T/VGH cells uPA protein and its enzymatic activity resulted unchanged in 5-aza-dC treated cells and inhibited in co-treated cells suggesting a major role of miR-193a toward its target uPA in these cells. In HepG2 cells at very low levels of uPA protein expression, the effects of 5-aza-dC with or without miR-193a were almost undetectable. Further investigations will examine whether this uPA overexpression may be due to DNA demethylation of its promoter or to other mechanisms in these 3 different HCC cells. Concerning the aggressive properties, our data indicated that the miR-193a ectopic expression and the 5-aza-dC treatment applied independently had a very low effect on inhibition of cell growth. Instead, in combination 5-aza-dC and miR-193a significantly decreased cell proliferation and migration abilities. These findings suggested that the ectopic expression of miR-193a may sensitize HCC cells to 5-aza-dC probably by acting on pathways not directly linked since they individually had a marginal effect. The combination of 5-aza-dC *plus* the oral multikinase inhibitor sorafenib, with or without miR-193a, did not show additional effects respect the use of low-dose sorafenib alone which had a more stable growth inhibitory effect in HCC cells. However, a recent phase I/II study confirmed the efficacy and safety of lower-dose 5-aza-dC based treatment that improved PFS (progression-free survival) and OS of 4 and 11 months respectively in patients with advanced HCC [43]. Therefore, it could be deduced that 5-aza-dC can be an option for the treatment of HCC, especially in patients not responsive to sorafenib or in patients developing resistance.

In summary, our results have shown that the miR-23b and miR193a down-modulation could significantly contribute to the molecular characterization of HCC diagnosis and that miR-193a may be a molecular prognostic factor for HCC patients; that DNA methylation mediates the miR-23b, and not miR-193a expression in human primary HCC. For biological activities of these miRs, only ectopic expression of miR-23b led to a significant proliferation inhibition of HCC cells and both miRs ectopic expression generated a strong inhibiting effect on migration abilities of HCC cells. Further, our finding of the increase of miR-23b expression in cultured HCC cells following 5-aza-dC treatment raises a novel issue to be elucidated on markers of treatment efficacy.

## MATERIALS AND METHODS

### Clinical samples of HCC

All human HCC samples and their corresponding PT non-tumour samples (resected 1–2 cm from the malignant tumour) were obtained from HCC patients undergoing surgical resection. Each biopsy specimen was obtained with the informed consent of patient under standard conditions of sampling and processing, as previously described and the procedures followed were in accordance with standards of ethics committee (NP1230) [23]. None of the patients had previously received therapeutic treatment. A total of 59 and 67 HCC patients were considered for miR-23b and miR-193a expression analyses, respectively, including the subsets of HCC patients evaluated beforehand: 17 for miR-23b [23] and 39 cases for miR-193a [22]. Each specimen was determined to be HCC or PT by pathological examination. The characteristics of the patients are described in Supplementary Table 2 and Supplementary Table 3.

### Cell cultures and treatments

The following human HCC cell lines were used in this study: HA22T/VGH (undifferentiated HCC-derived cells provided by Prof. N. D’Alessandro, University of Palermo, Italy), HepG2 (differentiated HCC-derived cells; ATTC HB-8065) and SKHep1Clone3 (SKHep1C3), selected from human HCC-derived cells (SKHep1: ATCC HTB-52). HA22T/VGH cells were maintained in RPMI-1640 (Life Technologies) while HepG2 and SKHep1C3 were maintained in Earle's MEM. Both media were supplemented with 10% foetal bovine serum and cells were grown at 37°C with 5% CO_2_. 5-Aza-2’-deoxycytidine (5-aza-dC; Sigma-Aldrich) was dissolved in DMSO (Sigma, St. Louis, MO) at the concentration of 50 mg/ml. To study DNA methylation levels, HCC cell lines were treated with 10 μM 5-aza-dC for 10 days (HA22T/VGH and HepG2) or for 5 days (SKHep1C3). Culture medium containing freshly added 5-aza-dC was replaced every 48 h. DMSO was added to cultures at 0.01% (v/v) as a solvent control. Sorafenib [*N*-(3-trifluoromethyl-4-chlorophenyl)-*N*-(4-(2-methylcarbamoylpyridin-4-yl) oxyphenyl) urea] was synthesized at Bayer Corporation (West Haven, CT). This compound was dissolved in DMSO and diluted with MEM to have a final concentration of 5 μM.

### Real-time quantification of mature miRNAs by stem–loop RT-PCR

The total RNAs were isolated from tissue samples and cells using TRIzol reagent (Invitrogen), according to the manufacturer's instructions. Mature miR-193a and miR-23b were quantitated by a two-step Taq-Man real-time PCR analysis, using primers and probes obtained from Applied Biosystems (Foster, CA, USA). cDNA was synthesized from total RNA (50 ng) in 15 μl reactions, using reverse transcriptase and the stem–loop primer for miR-193a (Applied Biosystems; PN 4427975), miR-23b (Applied Biosystems; PN 4373073) or RNU66 (internal control; Applied Biosystems; PN 4373382) contained in the TaqMan MicroRNA Reverse Transcription kit (Applied Biosystems, Foster, CA, USA). The reverse transcription reaction was performed by incubating the samples at 16°C for 30 min, followed by incubation at 42°C for 30 min and 85°C for 5 min. The q-RT PCR reaction (20 μl) contained 1.3 μl of reverse transcription product, 10 μl of Taq-Man 2× Universal PCR Master Mix and 1 μl of the appropriate TaqMan MicroRNA Assay (20×) specific for the miRNA targeted by the assay. The PCR mixtures were incubated at 95°C for 10 min, followed by 40 cycles at 95°C for 15 s and 60°C for 60 s. PCRs were performed in triplicate using the 7500 real time PCR system (Applied Biosystems). The expression of miR-193a and miR-23b was based on the ΔΔCT method, using RNU66 as an internal control. For each case the ratio (R= RQ_HCC_/RQ_PT_) between the relative levels in HCC and those in PT was assessed. The level of miRNAs expression was considered to be decreased for R values below 0.7 and increased for R values above1.3. When values were between 0.7 and 1.3, the miRNA expression was considered unchanged.

### DNA isolation and bisulfite modification

Genomic DNA was extracted from HCC cell lines (HA22T/VGH, HepG2 and SKHep1C3 treated or not with 5-aza-dC) and tissue samples (HCC and the corresponding PT), using the Wizard Genomic DNA Purification Kit (Promega) and the Blood Core Kit B (Qiagen), respectively, according to the manufacturer's instructions. To verify the integrity of genomic DNA, 100 ng of each sample was loaded on ethidium bromide-stained 0.8% agarose (w/v) gel and visualized as a high molecular weight single band. Bisulfite treatments of DNA were performed with EZ DNA Methylation Kit (Zymo Research), which allow purification and recovery of converted DNA in Zymo-Spin IC Columns. Bisulfite conversion was carried out on 1 μg of DNA for each sample, in the dark at 50°C for 12-16 hours.

### Methylation analysis of CpG sites associated with miR-193a and miR-23b by methylation-specific PCR (MSP)

Methprimer ( http://www.urogene.org/methprimer) was used to verify the presence of CpG islands in the 1.0 kb upstream sequences of miR-193a and miR-23b. To investigate the methylation of miRs CpG sites, methylation-specific PCR (MS-PCR) was performed. Bisulfite-converted DNA was amplified using primer sets specific for the methylated sequence (primers M) and the unmethylated sequence (primers U). The methylation-specific and unmethylation-specific primers were designed by MethPrimer ( http://www.urogene.org/methprimer/; Supplementary Table 4). Hot start-PCR was performed in the following way: a cycle of denaturation at 95°C for 5 min, followed by 40 (miR-193a) or 37 (miR-23b) cycles at 94°C for 30 s, 57°C for 30 s, 72°C for 30 s, and a final extension at 72°C for 3 min. MS-PCR products were visualized on ethidium bromide-stained 2% agarose (w/v) gel. The bands corresponding to methylated and unmethylated DNA were analyzed using a digital system (Gel-Pro Analyzer) and the integrated optical density (IOD) values were expressed in pixel. The percentage of methylation level in each sample was obtained using the following formula: IOD band Methylated/(IOD band M + IOD band Unmethylated) × 100.

### Transient transfection of miR-23b and miR-193a and 5-aza-dC/sorafenib treatments of HCC cells

Molecules of dsRNAs that mimic endogenous-miR-23b (5’-AUCACAUUGCCAGGGAUUACC-3’), miR-193a (5’-AACUGGCCUACAAAGUCCCAGU-3’) and pre-miRNA precursor negative control #1 (Life Technologies) were transfected into SKHep1C3 cells using Lipofectamine transfection reagent (Life Technologies), according to the manufacturer's instruction. To evaluate the effect on cell proliferation of miR-193a/miR-23b transfection in combination with 5-aza-dC treatment (or 5-aza-dC and sorafenib treatment), SKHep1C3 cells were pre-treated with 10 μM 5-aza-dC for 48 h and then trypsinized and seeded in 96-well plates (5 replicates for each experimental condition) at a density of 4 × 10^3^ cells/well in complete culture medium supplemented with 10 μM 5-aza-dC. Twenty-four hours after seeding, the cells were transfected with 50 and 100 nM pre-miR-193a into serum-free MEM containing 10 μM 5-aza-dC. After 5 h, the transfection medium was replaced with the complete medium supplemented with 5-aza-dC (or 10 μM 5-aza-dC combined with 5 μM sorafenib or 5 μM sorafenib alone, according to experimental conditions).

### Cell proliferation assay

The cellular proliferation was analysed using the CellTiter 96 Aqueous One Solution reagent (Promega, San Diego, CA, USA) after the pre-miR-193a/23b, pre-miR precursor negative control #1 (Life Technologies) transfections and 5-aza-dC/sorafenib treatments. The cells were seeded in 96-well plates (5 replicates for each experimental condition) at a density of 4 × 10^3^ cells/well in a complete cultured medium and 15 μl/well of sterile CellTiter reagent was added at the established time after transfection and/or 5-aza-dC/sorafenib treatments. The plates were measured 2 h after CellTiter addition using a microplate reader (EnSight, Perkin Elmer). The absorbance values at 490 nm were directly proportional to the number of living cells in the culture.

### *In vitro* scratch assay

120,000 SKHep1C3 cells were seeded in 24-well plate in complete medium, they were grown to 80% confluency and then transfected with 50 nM and 100 nM pre-miR-23b/pre-miR-193a or/and treated with 10 μM 5-aza-dC. After 24 h the transfection medium was replaced with fresh medium. When the cells reached the 100% confluence a scratch was made through the cell layer using a sterile micropipette tip. After washes with PBS, serum-free medium was added. The images of the wounded area were captured immediately after the scratch (T_0_) and 20 and 30 h later (T_1_ and T_2_) to monitor the cell migration into the wounded area. The migration abilities were quantified by measuring the area of the scratched regions using the ImageJ software. The experiment was performed twice.

### Western blot and zymography

The cellular extracts and media were collected from 24 h and 48 h cultures of SKHep1C3, HA22T/VGH and HepG2 cells under the following experimental conditions: cells treated with 10 μM 5-aza-dC for 5 days, cells treated with 10 μM 5-aza-dC and transfected with 100 nM miR-193a and control cells (DMSO and DMSO + Lipofectamine, respectively). To evaluate the levels of MET and GAPDH expression, cells were lysed in 0.05% SDS and constant amounts of protein were loaded on 4-12% Novex NuPAGE Bis/Tris gels (Invitrogen) under reducing conditions. To detect uPA expression, proteins contained in the conditioned media were separated using 8% SDS polyacrylamide gels under non-reducing conditions and electro-transferred on nitrocellulose membranes (NM). The following primary antibodies were used: rabbit anti-human MET (1:1000 in 0.3% BSA; Santa Cruz Biotecnology), mouse monoclonal antibodies anti-GAPDH (1:500 in 1% BSA; Merck Millipore) and rabbit anti-human uPA (1:1000 in 1% BSA; Technoclone). Primary antibodies were stained using alkaline phosphatase-conjugated anti-rabbit or anti-mouse IgG (1:7500 in 0.3% BSA; Promega). The results of the immunoreaction were detected with Western Blue Stabilized Substrate for Alkaline Phosphatase (Promega). Protein bands were visualized and analyzed using the Gel-Pro Analyzer software and the integrated optical density (IOD) values were expressed in pixels. uPA activity was evaluated in zymography in which NMs were overlaid onto casein agar containing 2 μg/ml human plasminogen (Technoclone).

### Statistical analysis

Statistical analyses were performed using GraphPad Prism 6.01. Student's t-test was used to determine the differences of miRNAs expression and DNA methylation between HCC and PT samples and between 5-aza-dC treated cells and their control, and for uPA and MET expression. Receiver-operating curves (ROC) were conducted to determine ability of miR-23b and miR-193a to discriminate between tumor and PT tissues. For survival curves, Kaplan-Meier analyses were conducted using 30 cases. Analysis of variance (ANOVA) followed by post-hoc Tukey test was applied to discern significant differences in the proliferation assays. Correlation between miR-23b/193a expression levels and clinical and pathological characteristics was evaluated using Chi-square test. Data were considered significant when P-value ≤ 0.05.

## SUPPLEMENTARY MATERIALS FIGURES AND TABLES




