# Supplementary material for: Clinical and biological significance of miR-23b and miR-193a in human hepatocellular carcinoma
Source: Oncotarget. 2016 Dec 28;8(4):6955–69. doi: 10.18632/oncotarget.14332 (PMC5351682; doi:10.18632/oncotarget.14332)
Supplement: Supplementary file 1 [file oncotarget-08-6955-s001.pdf]

Clinical and biological significance of miR-23b and miR-193a in human hepatocellular carcinoma

SUPPLEMENTARY FIGURES AND TABLES

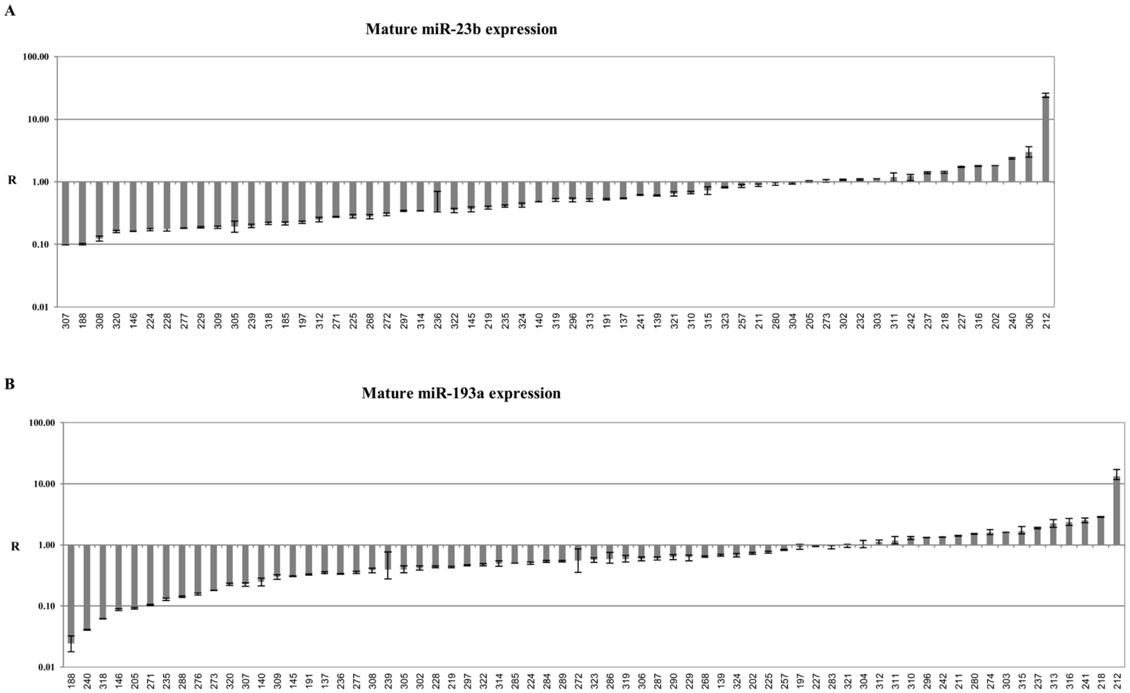

Supplementary Figure 1: Expression of mature miR-23b and miR-193a by qPCR in HCC and PT tissues from biopsies of HCC patients. miR-23b **A.** and miR-193a **B.** profiling was detected by stem-loop qPCR in 59 and 67 HCC patients, respectively. The histograms indicate the R-values (RQ_{HCC}/RQ_{PT}) for each sample tested. The histograms are ordinated by increasing R.

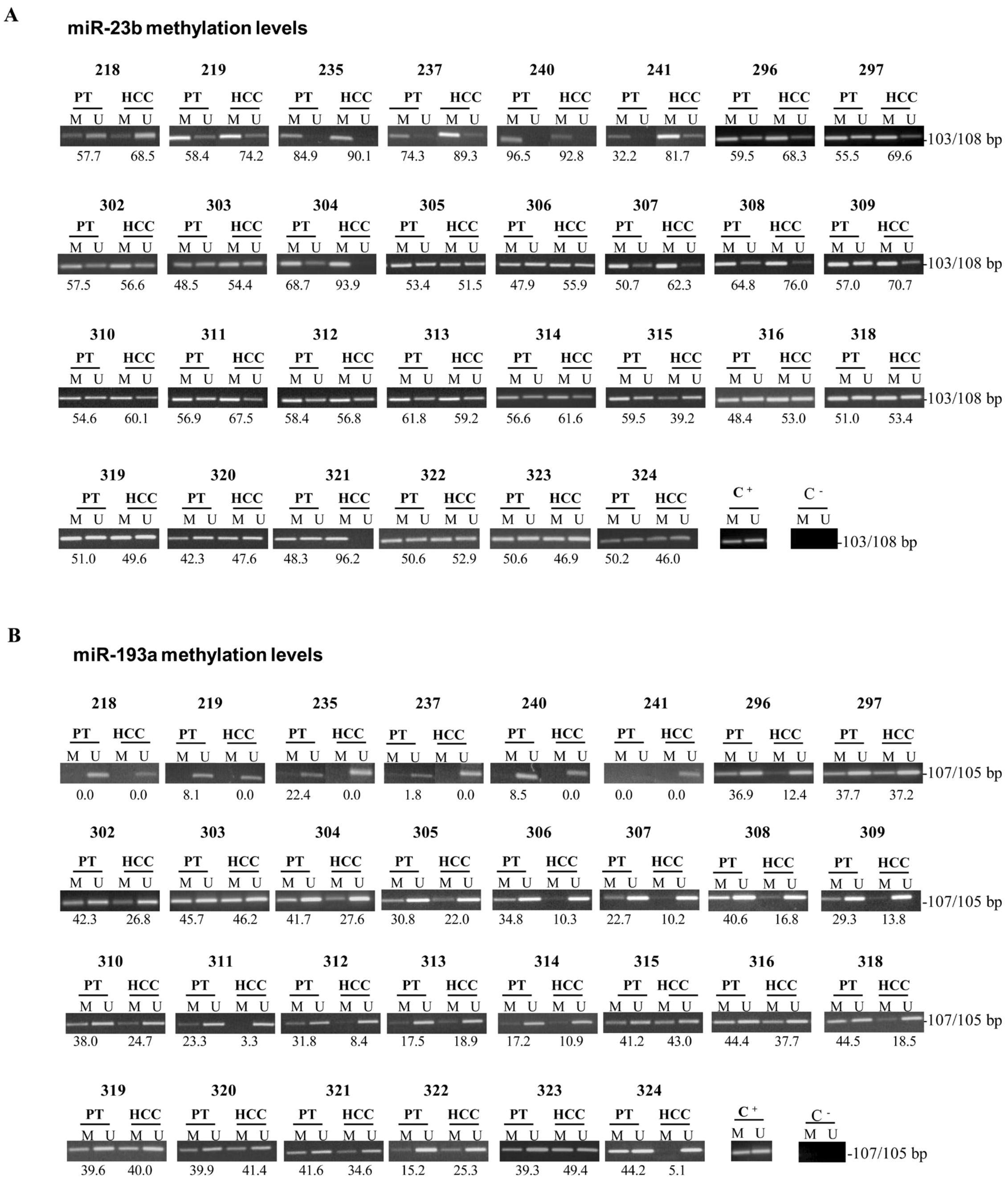

Supplementary Figure 2: Methylation levels of miR-23b and miR-193a detected by MS-PCR in 30 HCC and PT tissues from biopsies of HCC patients. The methylation profiling of miR-23b **A.** and miR-193a **B.** was detected by Methylation-Specific PCR (MS-PCR) in 30 HCC patients. The percentage of DNA methylation (IOD M/IOD M + IOD U x 100) is indicated for each sample (IOD = Integrated Optical Density). C^+: positive controls for methylated and unmethylated DNA, respectively. C^-: negative controls.

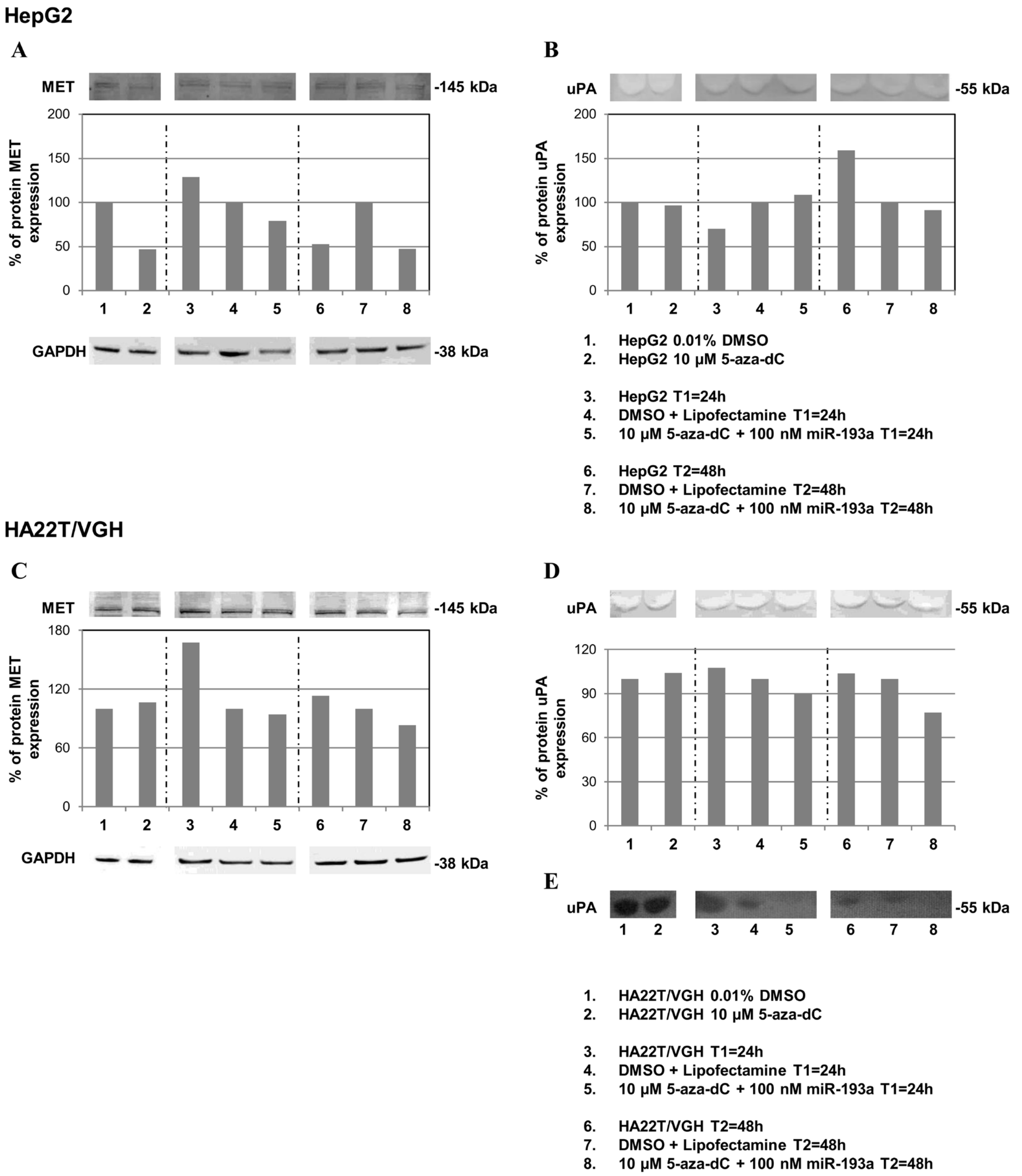

Supplementary Figure 3: MET and uPA protein expression levels in HepG2 and HA22T/VGH cells treated with 10 µM 5-aza-dC and transfected with 100 nM miR-193a. HepG2 and HA22T/VGH cells were treated with DMSO (vehicle) and 10 µM 5-aza-dC or treated with 10 µM 5-aza-dC in combination to 100 nM miR-193a transfection. Western blot was performed to detect MET and GAPDH expression **A-C.** on cell extracts and uPA expression **B-D.** on conditioned media. The uPA enzymatic activity was shown in HA22T/VGH cells **E.** and was undetectable in HepG2 cells (data not shown).

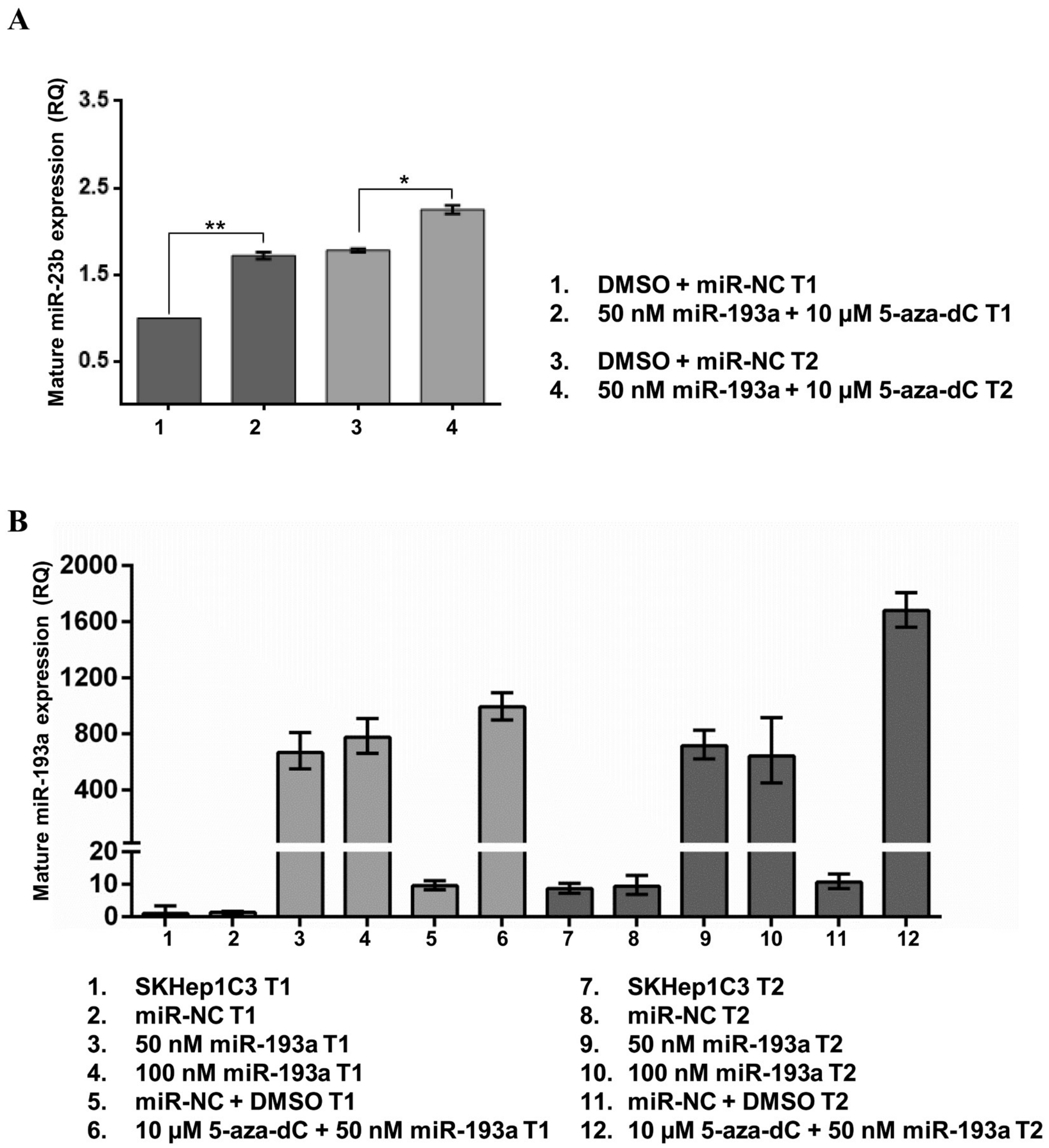

Supplementary Figure 4: Evaluation of mature miR-23b and miR-193a levels by qPCR in SKHep1C3 cells after 5-aza-dC treatment and miR-193a ectopic expression. miRs expression levels were detected by stem-loop qPCR in SKHep1C3 cells treated with 10 µM 5-aza-dC and transfected with 50 nM miR-193a and relatives controls. **A.** 5-aza-dC treatment significantly increased miR-23b expression also in combination with miR-193a transfection and relative controls at 24h and 48h after transfection. The histograms represent the mean of two experiments and bar are SEM values; **p<0.001 and *p<0.05 in t-test analysis. **B.** Evaluation of mature miR-193a level in cells transfected with miR-193a in combination or not to 5-Aza-dC treatment. The histograms represent RQ (relative quantification) values; bars are maximum and minimum RQ values.

Supplementary Table 1: Correlation between miR-23b (A) and miR-193a (B) expression levels and clinical and pathological characteristics in 59 and 67 HCC patients

A	Number of patients	High expression	Low expression	p-value[#]
**Gender**				
Male	41	15	26	0.510
Female	18	5	13	
**Differentiation grade**				
G1 - G2	30	10	20	0.912
G3 - G4	23	8	15	
**Cirrhosis**				
No	22	8	14	0.475
Yes	33	9	24	
**Hepatitis B infection**				
Negative	39	13	26	0.894
Positive	19	6	13	
**Hepatitis C infection**				
Negative	29	7	22	0.162
Positive	29	12	17	

B	Number of patients	High expression	Low expression	p-value[#]
**Gender**				
Male	46	15	31	0.953
Female	21	7	14	
**Differentiation grade**				
G1 - G2	37	12	25	0.971
G3 - G4	25	8	17	
**Cirrhosis**				
No	22	7	15	0.426
Yes	41	14	27	
**Hepatitis B infection**				
Negative	45	13	32	0.262
Positive	21	9	12	
**Hepatitis C infection**				
Negative	34	12	22	0.728
Positive	32	10	22	

[#]P-values were obtained using Chi-square test.

Supplementary Table 2: Clinical and pathological characteristics of HCC patients enrolled for the evaluation of miR-23b and miR-193a expression

**Parameters**	**Subset miR-23b expression**	**Subset miR-193a expression**
**Total number of patients enrolled**	59	67
**Age**, mean (range)	67.4 (38-82)	68 (38-82)
**Sex**, No. (%)		
Male	41 (69)	46 (69)
Female	18 (31)	21 (31)
**Cirrhosis**, No. (%)		
Yes	33 (56)	41 (61)
No	22 (37)	22 (33)
Not available	4 (7)	4 (6)
**Hepatitis virus infection**, No. (%)		
HBV	14 (24)	16 (24)
HCV	24 (41)	27 (40)
HBV/HCV positive	5 (8)	5 (7)
HBV/HCV negative	15 (25)	18 (27)
Not available	1 (2)	1 (1)
**Differentiation grade**, No. (%)		
G1	8 (14)	7 (10)
G2	22 (37)	30 (45)
G3	21 (36)	24 (36)
G3/G4	2 (3)	1 (1)
Not available	6 (10)	5 (7)

Supplementary Table 3: Clinical and pathological characteristics of the studied cohort of HCC patients

See Supplementary File: 1

Supplementary Table 4: Primers used in MS-PCR to examine DNA methylation level of miR-23b and miR-193a

Primer	Sequence from 5' to 3'
**miR-23b**	
**Forward M primer**	ATTATGTGTGTTAGGAAAGGGAAAC
**Reverse M primer**	CTATATACCGCCAACCCGTC
**Forward U primer**	ATTATGTGTGTTAGGAAAGGGAAAT
**Reverse U primer**	ACAATCTATATACCACCAACCCATC
**miR-193a**	
**Forward M primer**	GTCGAGTTGAGCGTAGGTAATC
**Reverse M primer**	CGAATAAAACGCAAAAATTATACG
**Forward U primer**	GTTGAGTTGAGTGTAGGTAATTGA
**Reverse U primer**	CAAATAAAACACAAAAATTATACAC
