# Supplementary material for: Clinical and biological significance of miR-23b and miR-193a in human hepatocellular carcinoma
Source: Oncotarget. 2016 Dec 28;8(4):6955–69. doi: 10.18632/oncotarget.14332 (PMC5351682; doi:10.18632/oncotarget.14332)
Supplement: Supplementary file 2 [file oncotarget-08-6955-s002.doc]

| **Supplementary Table S3:** Clinical and pathological characteristics of the studied cohort of HCC patients | | | | | | |  |
| --- | --- | --- | --- | --- | --- | --- | --- |
| **Case** | **Gender** | **Years** | **Grading** | **TNM** | **Background disease** | **HBV** | **HCV** |
| 137 | M | 69 | G3 | T3bN0M0 | active cirrhosis | - | + |
| 139 | M | 65 | G2 | T2N0M0 | active cirrhosis | + | + |
| 140 | M | 69 | G2 | T1N0M0 | aspecific reactive hepatitis | - | + |
| 145 | M | 65 | G2 | T1N0M0 | steatotic hepatitis with portal and periportal fibrosis | - | - |
| 146 | M | 61 | na | na | na | - | + |
| 185 | M | 66 | G1 | na | active chronic hepatitis with steatosis of moderate level | + | - |
| 188 | M | 73 | G1 | T3bN0M0 | cirrhosis with active chronic hepatitis | + | + |
| 191 | F | 63 | G1 | T1N0M0 | cirrhosis with active chronic hepatitis | - | + |
| 197 | M | 70 | G2 | T1N0M0 | cirrhosis with micro- and macrovescicular steatosis | - | - |
| 202 | M | 76 | na | na | na | na | na |
| 205 | M | 73 | G2 | T1N0M0 | active chronic hepatitis | - | + |
| 211 | M | 51 | G2 | T1N0M0 | cirrhosis with active chronic hepatitis with foci of macrovescicular steatosis and presence of iperplastic and regenerative macronodules | + | + |
| 212 | M | 64 | G2 | T1N0M0 | cirrhosis with active chronic hepatitis | + | + |
| 218 | M | 64 | G2 | T2N0M0 | cirrhosis with active chronic hepatitis | + | - |
| 219 | M | 57 | G1 | T1N0M0 | cirrhosis with active chronic hepatitis | + | - |
| 224 | M | 55 | G3 | T3bN0M0 | cirrhosis with active chronic hepatitis | + | + |
| 225 | M | 49 | G3 | T3bN0M0 | microvescicular steatosis; focal lipofuscinosis; cholestasis | - | - |
| 227 | F | 72 | G2/G3 | T1N0M0 | cirrhosis with active chronic hepatitis | - | + |
| 228 | M | 59 | G2 | T1N0M0 | active chronic hepatitis of severe level with necrosis and bridging porto-portal fibrosis ( HBsAG) | + | - |
| 229 | F | 79 | G2/G3 | T3bN0M0 | cirrhosis with active chronic hepatitis | - | + |
| 232 | M | 76 | na | na | active chronic hepatitis with porto-portal necrosis and fibrosis versus cirrhosis | - | + |
| **Case** | **Gender** | **Years** | **Grading** | **TNM** | **Background disease** | **HBV** | **HCV** |
| 235 | F | 82 | G3 | T2N0M0 | cirrhosis with active chronic hepatitis | - | + |
| 236 | F | 76 | G1 | T1N0M0 | cirrhosis with active chronic hepatitis | - | + |
| 237 | M | 68 | G2/G3 | T1N0M0 | mildly active chronic hepatitis | - | + |

| 239 | M | 66 | G2/G3 | na | cirrhosis and active chronic hepatitis | - | - |
| --- | --- | --- | --- | --- | --- | --- | --- |
| 240 | M | 71 | G3 | T3bN0M0 | active chronic hepatitis with necrosis and bridging and portal fibrosis | + | - |
| 241 | F | 38 | G2 | T3N0M0 | reactive hepatitis | + | - |
| 242 | F | 63 | G2 | T2N0M0 | active chronic hepatitis with focal fibrosis and with bridging porto-portal fibrosis | - | + |
| 257 | M | 69 | G1/G2 | T1N0M0 | cirrhosis and hemochromatosis | - | - |
| 268 | F | 68 | G1 | T1N0M0 | cirrhosis with active chronic hepatitis | + | - |
| 271 | F | 71 | G2/G3 | T1N0M0 | cirrhosis with active chronic hepatitis | - | + |
| 272 | M | 65 | G1 | T2N0M0 | cirrhosis with active chronic hepatitis and macro-and microvescicular steatosis (30% of parenchyma) | - | - |
| 273 | M | 73 | G2 | T1N0M0 | cirrhosis with active chronic hepatitis and mild macro- and microvescicular steatosis | - | + |
| 274 | F | 81 | G2 | T1N0M0 | mildly active chronic hepatitis with micro- and macrovescicular steatosis (30% of parenchyma) | - | - |
| 276 | M | 72 | G2 | T1N0M0 | cirrhosis with active chronic hepatitis | - | + |
| 277 | F | 75 | G2 | T2N0M0 | mildly active chronic hepatitis | - | - |
| 280 | F | 74 | G2/G3 | T1N0M0 | active cirrhosis | - | + |
| 283 | M | 78 | G2 | T1N0M0 | mildly active chronic hepatitis | + | - |
| 284 | M | 76 | G2 | T1N0M0 | active chronic hepatitis | + | - |
| 285 | M | 77 | G2 | T2N0M0 | active chronic hepatitis with necrosis ponte-portale of moderate/severe level | - | + |
| 286 | M | 69 | G3 | T4N0M0 | active cirrhosis | - | + |
| 287 | M | 63 | G2 | T2N0M0 | active cirrhosis | - | - |
| 288 | F | 64 | G2 | T1N0M0 | active cirrhosis | - | - |
| 289 | M | 75 | G2 | T1N0M0 | cirrhosis with iperplastic-displastic macronodules | - | + |
| 290 | M | 65 | G2/G3 | T1N0M0 | cirrhosis with active chronic hepatitis | + | - |
| **Case** | **Gender** | **Years** | **Grading** | **TNM** | **Background disease** | **HBV** | **HCV** |
| 296 | M | 67 | G3 | na | active cirrhosis | + | - |
| 297 | M | 73 | G3/G4 | na | active cirrhosis | + | - |
| 302 | F | 61 | G2 | na | cirrhosis with active chronic hepatitis | - | - |
| 303 | M | 76 | G3/G4 | na | active cirrhosis | - | - |
| 304 | M | 70 | G2 | na | cirrhosis with active chronic hepatitis | - | + |
| 305 | F | 76 | G3 | na | chronic hepatitis | - | + |
| 306 | M | 48 | G3 | na | hepatitis chronic and steatosis of second level | - | + |
| 307 | M | 64 | G2 | na | steatosis of first level | - | - |
| 308 | M | 73 | G2 | na | active cirrhosis with steatosis of second level | - | - |
| 309 | M | 68 | G2/G3 | na | mildly active chronic hepatitis with bridging porto-portal fibrosis | - | + |
| 310 | F | 71 | G1 | na | cirrhosis with active chronic hepatitis | - | + |
| 311 | M | 71 | G2/G3 | na | mildly active chronic hepatitis with focal fibrosis and bridgin porto-portal necrosis | + | - |
| 312 | M | 69 | G3 | na | hepatitis with steatosis and bridging porto-portal fibrosis with poorly differentiated cholangiocarcinoma component | + | - |
| 313 | M | 61 | na | na | cirrhosis of the liver extensively necrotic | - | - |
| 314 | F | 64 | G2 | na | active cirrhosis | - | - |
| 315 | M | 66 | na | na | cirrhosis with active chronic hepatitis | - | + |
| 316 | F | 71 | G2 | na | na | + | - |
| 318 | F | 71 | G2 | na | cirrhosis with active chronic hepatitis | - | + |
| 319 | M | 72 | G2 | na | cirrhosis with active chronic hepatitis | - | - |
| 320 | M | 71 | G2/G3 | na | severe active chronic hepatitis with necrosis and bridging porto-portal fibrosis versus cirrhosis | - | - |
| 321 | M | 71 | G3 | na | cirrhosis with active chronic hepatitis, signs of portal hypertension. Presence of a small biliary adenoma | - | + |
| 322 | M | 54 | na | na | aggressive chronic hepatitis, increase of the branches of the portal vein | + | - |
| 323 | F | 78 | G3 | na | na | - | + |
| 324 | F | 78 | G3 | na | cirrhosis with active chronic hepatitis | + | - |

^*^ na: not available data
